# Actinomycosis1Read at a meeting of the Veterinary Medical Society of the Ontario Veterinary College.

**Published:** 1896-02

**Authors:** John Latham

**Affiliations:** Veterinary Student, Ontario Veterinary College, Ontario, Can.


### ACTINOMYCOSIS.[1]

By JOHN LATHAM,
VETERINARY STUDENT, ONTARIO VETERINARY COLLEGE, ONTARIO, CAN.

Actinomycosis is a granulomatous disease caused by the bacillus actinomycosis, which is a minute vegetable fungus said to consist of a conical mass of branched club-like filaments springing from a single cell and bearing spores on their terminal branchlets. Some authorities claim that this fungus is closely allied to that which produces the green mould on damp leather; others think it closely allied to the fungus causing rust and smut in cereals and grasses.

Pathological research is yearly demonstrating more clearly

[1] Read at a meeting of the Veterinary Medical Society of the Ontario Veterinary College.

the mighty part played by microscopic vegetable organisms in the production of disease in plants and animals, the extreme minuteness and retentive vital powers of such relentness foes making prevention and treatment alike difficult and unsuccessful. The use of the microscope has shown us that tuberculosis, anthrax, swine-plague, fowl-cholera, leprosy, etc., are due to microphytes, and among them we include actinomycosis.

How does this germ gain entrance into the animal body? The ox tribe seems to be the most susceptible to infection, and it is generally supposed that the spores gain entrance with the food or water, or may float in the air, and so be inhaled, the disease in some instances having been located in the trachea and lungs. An abrasion of the mucous membrane is considered necessary before the fungus can produce the disease. The necessary abrasion might be produced in some cases by the agent by which it gains entrance, such as barley, acorns, or other rough food.

*Symptoms.* Having entered the animal and found a suitable culture-ground, which it appears most commonly to find in the region of the mouth, the parasite commences to grow and multiply, and as it does so it destroys to a greater or less degree the natural tissue, with the result that inflammation of a chronic form is set up, and, Nature endeavoring to repair the damage, new tissue, generally of a fibrous nature, is formed. This when in connection with the jaw shows a tendency to become ossified, hence we have an increase in size and weight of the part, while the original tissue may be almost entirely destroyed. If the tongue be the part involved, it becomes enlarged and very hard, loosing its pliability, and in virtue of this condition has been called wooden tongue.

After a variable period the morbid process may develop an abscess, and in the resulting discharge will be noticed minute yellow granules, which, when properly prepared and magnified, are found to consist of the actinomycosis cluster in the centre, surrounded by several layers of cells, and the whole enclosed in a fibrous capsule. Pathological changes may go on to quite an extent, and so long as septic germs do not find an entrance, and the functions of the vital organs are not seriously interfered with, the animal may not evince any signs of serious constitutional disturbance, but may continue in good flesh. If, however, the processes of mastication, deglutition, or respiration be interfered with to any extent the animal will speedily run down, lose flesh, and if left to itself soon die.

The question has been asked in our meetings how long will an animal live when attacked with this disease. I have seen a beautiful two-year-old heifer, weighing 1700 pounds, reduced to a skeleton in less than a year, commencing with a single lump, like a walnut, on the lower maxillary bone, followed by others on the superior maxilla, the radius, and dorsal vertebra. Dr. Rayen, a former graduate of this College, now State Veterinarian of Tennessee, who held the post-mortem, and had the benefit of a good microscope, found the disease in nearly all the internal organs. Her stall companion, which had licked the open abscesses on the head of this heifer, was operated on the same day, having a lump as large as a goose-egg on the lower maxillary bone, which was removed, scraped, and treated antiseptically. In four weeks she was sent to pasture and is still in the breeding-herd, showing no further sign of disease.

Another native cow that also made a practice of licking the first heifer was similarly affected, and her calf, when only seven weeks old, showed a well-defined tumor on the lower jaw. Both cow and calf, being of little value, were not treated, this being as early as 1887, when the iodide of potassium treatment was unknown.

In August of 1891 I had in charge a pure-bred short-horn which developed a lump on the jaw, also a hard, fibrous tumor on the side of the trachea below the larynx. This heifer was treated with iodide of potassium in ʒij doses, morning and evening for a week, when signs of iodism began to show, and treatment was withheld for six days. When renewed iodism came on in five days, a rest of seven days was given, when only half the dose was given for a week, with the result that the tumor on the neck had almost disappeared, while the one on the maxilla had increased in size. During this treatment the animal had the best of care, nice, soft, nourishing diet, liberally salted, thereby getting the full benefit of the iodine without the usual constipation attending its continued use. I then operated on the jaw after Dr. Rayen's plan, the wound healing nicely, and the heifer passed muster in the Chicago Stockyards in December.

Of the 185 animals suffering from this disease in an advanced stage treated by Dr. Salmon on iodide of potassium, 131 responded to treatment. Among them were placed 21 healthy subjects, which after four months' exposure showed no signs of disease, and post-mortem after slaughter confirmed the diagnosis. On the other hand, it is a well-known fact that in a herd of pure-bred Hereford cattle maintained in Illinois, 75 had become more or less affected in the short space of four years.

At this writing, opinion is still divided as to whether or not this is a contagious disease. While not considering it highly so, I would by all means recommend isolation of affected animals, especially after suppuration is established.

*Treatment.* In case of valuable animals being affected, excision of the tumors, followed by the iodide of potassium, is the quickest and by far the most satisfactory means of dealing with this disease for both the owner and veterinarian, while for animals of less value we may be governed by circumstances.

The *modus operandi* of iodine in combating this disease is not fully established, some authorities advancing the rather plausible theory that in order to live and increase in the animal body it is found to be necessary that the parasite be surrounded by a certain amount of liquefied tissue; and in virtue of its very strong absorbent properties, especially upon pathological accumulations, the drug thus destroys the germ through removal of the necessary culture-medium.

Is the flesh fit for human food? There are on record nearly 300 cases of actinomycosis in the human family. Dr. William Home, of Janesville, Wis., who has made a special study of this disease, records a case where seven persons were poisoned by eating the flesh from the fore-quarter of a cow suffering from actinomycosis. Knowing this, and also that the spores of actinomycosis are only one-quarter the size of a red blood-corpuscle, and that these spores have been found in all parts of the circulation, unless such flesh is subjected to a heat of 212° (boiling-point), it must be dangerous. In view of the cheapness of prime beef, and value of human life, the rendering-tank seems the proper disposition of affected subjects.

---

Dr. Robert W. Ellis, of New York City, reports a case of tetanus successfully treated with tetanus antitoxin.

